# Clinical Humanities; informal, transformative learning opportunities, where knowledge gained from Humanities epistemologies is translated back into clinical practice, supporting the development of professional autonomy in undergraduate dental students

**DOI:** 10.15694/mep.2018.0000163.2

**Published:** 2018-11-14

**Authors:** Flora Smyth Zahra

**Affiliations:** 1 King's College London Dental Institute

**Keywords:** Transformative learning, Student personal and professional identity formation, Knowledge translation from Humanities epistemologies to clinical practice, critical reflection, socio-cultural health determinants

## Abstract

This article was migrated. The article was marked as recommended.

Nurturing the successful professional and personal development of undergraduate dental students is the fundamental obligation of their Clinical teacher. Considering this within a framework of the various models of professional development provides an appropriate lens to analyse in depth the informal learning that takes place within the workplace of the dental clinics. In an effort to then address some of the perceived shortcomings of the existing dental curriculum an innovative arts and humanities intervention is subsequently described which in providing alternative informal learning opportunities appears to be increasing student clinical capabilities and humanistic skills, thus enhancing their professional and personal development and improving their capacity to deliver holistic patient care.

## Professional development in dental education

### Models of professional development

University learning is usually regarded as rigorous, intellectual engagement with an academic discipline and associated with hours of often solitary studying on the road to knowledge acquisition. Professional learning and development however, inherently aligned to both the definition of a profession as an occupation where one publicly declares a skill and professionalism, the action of participating according to a set of doctrines adopted by others with a similar skill set, are by nature social and involve learning within the workplace. Of the many models of professional development, ( Kennedy, 2005) has identified nine according to their key characteristics which she then further defines as either; transmission, transitional or transformative according to the type of knowledge developed and their purpose. She suggests that as one moves from transmission models such as award - bearing or cascade models through the more transitional; coaching/mentoring and communities of practice methods towards the transformative; action research and an agenda focussed transformative practice model which integrates aspects from the entire range, there is a shift in power from the regulators and standard-setters to the learner providing them with increased professional autonomy. Although in her introduction, Kennedy hopes that her analysis will provide understanding of ‘the nature of professional knowledge and professionalism itself’ this is not made entirely explicit as she does not proffer any further elaboration, however she does agree with ( Eraut, 1994) that the context of professional learning is crucial within the policies of the institution itself, the formal academic curriculum and work based practice, additionally highlighting the importance of informal learning opportunities.

### Dental undergraduate education and student professional and personal development

The study of Dentistry is a combination of both intellectual engagement and professional learning. The clinical teaching of undergraduates is by definition work place based and involves helping students integrate academic, theoretical knowledge with the practical and experiential. The University degree is accredited for professional status and as such the regulator states that ‘Students must demonstrate during their education and training that they have the knowledge, skills and attitudes expected of a registered dentist’ ( GDC, 2015). The requirements are therefore extremely high, non-negotiable and as has been said of graduating teachers but equally could be applied to novice dentists, ‘Excellent teachers do not emerge full blown at graduation [...] Instead; teachers are always in the process of ‘becoming.’ Given the dynamics of their work, they need to continuously rediscover who they are and what they stand for [...] through deep reflection about their craft’ ( Nieto, 2003).

It is therefore fundamental to teach the students of the complex realities of real life clinical practice providing them with the necessary skills to develop learning strategies for the entirety of their practising lives. Kennedy’s framework ( Kennedy, 2005) affords a useful lens to examine the professional development of undergraduate dental students so inextricably linked also to their personal development. Of particular relevance is the transitional to transformative end of the spectrum; the community of practice and transformative models critically linked to both empowerment and increased professional autonomy. Within these models of professional development, opportunities exist to address the impact of the informal and hidden curricula, often erroneously regarded as being of lesser importance than the formal aspects of learning but in fact inextricably linked to the delivery of good patient care and also acknowledging that a ‘love of informal learning’ is a ‘key characteristic of lifelong learners’ ( Coffield, 2000).

### Informal learning ‘situated’ in the dental clinics

Dental students of King’s College London are introduced to learning in the clinical setting as members of one of four teams from the first term of year one. Initially these short visits, interspersed between formal lectures provide opportunities to carry out simple cross- infection control procedures supervised by the nursing staff and enable informal contact between peers from other year groups and clinical teachers. Over the subsequent four years, students spend increasingly longer periods of time on the clinics treating their own patients under supervision across as wide a range of dental specialities as they will encounter in practice, their skill sets constantly evolving and improving. This is the type of social learning environment advocated by ( Dewey, 1938), ( Vygotsky, 1978) and later ( Lave and Wenger, 1991), where the students, encouraged to enquire and problem solve, construct their own knowledge, creating meaning from real life experience associated with the ‘context-specific’ transformative and community of practice models Kennedy (2005) describes. In contrast to the often de-contextualised formal lectures where the students are passive listeners, ‘situated’ (Lave and Wenger, 1998) within the community of practice of the clinic their learning ‘takes place through the relationships between people and connecting prior knowledge with authentic, informal, and often unintended contextual learning’ ( Northern Illinois University, 2012).

Transformative learning encourages self-examination through ‘discourse and critical reflection’ ( Mezirow, 1991) promoting change in beliefs and construction of new knowledge. As Kennedy makes clear, for this informal model of professional development to have maximum capacity for transformation, the ‘issue of power’ ( Kennedy, 2005) needs to be addressed. The role of the Clinical teacher is less of a ‘transmitter of knowledge’ (Lave and Wenger, 1998) and more of a facilitator, helping the students develop the necessary skills, empowering their move from the periphery of the clinic in year one to becoming active participants, practising dentists, colleagues within the community of practice.

In the context of medical education, this situated learning can ‘be conceived as a ‘cultural phenomenon’ is ‘characteristically collaborative’ and within the clinical setting the informal curriculum ‘unfolds with opportunities for engagement’ ( Swanwick, 2005). This stance is in accordance with that of Lave and Wenger who argued that ‘engagement is a fundamental prerequisite for informal learning’ ( Swanwick, 2005).

### Informal learning: - implicit, explicit and tacit

Working together as part of the clinical team, delivering care to patients, the actual mechanism of this informal learning through praxis is complex. Some of the learning on the clinic is deliberate but much of it is incidental, related to the hidden curriculum, the norms and values of the profession, often acquired implicitly, ‘independently of conscious attempts to learn and the absence of explicit knowledge about what was learned’ ( Reber, 1967). Tacit knowledge, ‘knowing more than we can tell’ (Polyani, 1967) plays a fundamental part in the social interactions between clinical teachers and students on the clinic. On the one hand, the teachers with procedural knowledge literally embedded in their hands over many years try to make their intuitions and that which has become tacit, explicit to the students. On the other, the students develop the capability over time through their own interactions to be able to respond to clinical scenarios as a matter of course and thereby construct their own tacit knowledge from what was explicit. Previous work has viewed this transformational learning in dentistry as passing through a series of thresholds and attempts to ‘visualise’ the development of dental expertise have employed the use of concept maps. ( Kinchin, Cabot and Hay, 2010)

### Clinical Humanities - providing new informal learning opportunities to further dental student personal and professional development

Amongst what has been referred to as ‘the essence of professionalism’ within medical education and equally may be applied to the education of dentists are the attributes of ‘altruism, accountability, duty, integrity, respect for others and lifelong learning,’ ( Gordon, 2003) who then suggests that a student professional and personal development curriculum that attempts to inculcate these attributes should include; ‘communication skills, humane care or humanism, self-care, ethics and a medical humanities component’ ( Gordon, 2003). Cohen has gone further and argues that, ‘humanism is the passion that animates professionalism’ and that developing student humanistic skills through the role modelling of caring practice and ‘serious engagement with the medical humanities’ is the ‘key to fostering their professionalism’ ( Cohen, 2007). Others agree that by ‘marrying the applied scientist to the medical humanist’ a good doctor ‘can be made’ ( Hurwitz, 2002). An ideal dental education should promote, ‘critical thinking, lifelong learning, a humanistic environment, scientific discovery and integration of knowledge, evidence based oral health care, assessment, faculty development, and the health care team’ ADEA, 2006). As has been acknowledged, ( Gordon, 2003) many medical schools now include medical humanities components in their curricula in an effort to both improve not only clinical reasoning and interpretative skills but also to foster student professional and personal development. Although dental students face many of the same pressures and concerns as medical students, review of the literature base suggests that there has been virtually no such progress within dentistry. In an effort to address this, over the past three years I have been piloting, embedding and developing new courses at King’s with Harvard Dental being an early adopter, encouraging the students to consider the practice of dentistry from the perspective of the humanities, which I have called Clinical Humanities following Shapiro, that is to say ‘the study of the humanities that is strongly linked to praxis in fields such as medicine, nursing, occupational therapy [and dentistry] that serve those who are ill, incapacitated and suffering’ (Shapiro, 2014).

A diverse range of themes allows the students to experience a wide range of knowledge including; History of Medicine, Philosophy, visual and performing arts, ceramics, surgery as craft and narrative in film. A core principle is, namely that each session is delivered jointly between clinicians and humanities experts to maintain relevance not only to praxis but to support new knowledge making or *phronesis.* Session examples include:-

**History of Medicine and Philosophy** Insights from history can teach students about inequalities in access to care, the importance of social health determinants, that the burden of disease, efficacy and how medicine is practised, changes over time amidst the given social and political context. This initial session commences with a guided walk through Guy’s campus discussing the students’ own context and stopping by the statue of John Keats and the plaque to Wittgenstein before continuing to the Old Operating Theatre and Herb Garret in St Thomas’ Street. The notion of different epistemologies is made explicit to the students together with joint presentations by the curator of the museum and myself that explore; the context of dentistry in the history of surgery, the meaning of ambiguity, the inherent uncertainty of clinical practice, social determinants of health, person centred care and how aspects of practice, care and consent change over time.

**Art, close observation and free writing** This second session, led jointly by myself and an art historian colleague at The Courtauld Institute, King’s Strand campus, explores ways of seeing, problem solving as a team, the dental examination, and building rapport with patients from the initial encounter, smile aesthetics, discrimination, symmetry, golden ratio, the balance of tones and textures and ambiguity through close observation of two famous works of art; Lucas Cranach the Elder ‘s painting of Adam and Eve, 1526, and Edward Manet’s, ‘A Bar at the Folies-Bergère,’ 1882. The session ends with a free writing exercise.

**Close listening, communication and performance** This is a performance workshop at the Dental Institute with a professional actor alongside myself and another clinical teacher, that considers the importance of close listening, allowing patients time to ‘tell their story,’ and the relation between practice and performance, dealing with nerves, good communication techniques, voice, posture, non -verbal communication, accurate writing and considering what happens when things go wrong in clinical practice.

**Ambiguity in Film** As a narrative form, film can be a particularly powerful tool for clinicians to experience an enormous gamut of human interaction, experience of illness, analysis of patient clinician relations, life stories, different cultures and a multitude of different perspectives and ambiguities. Students are asked to watch two foreign language subtitled films in advance of this session held in the main lecture theatre of the dental school and then spend the session considering short sections of both films together with myself and a professional screenwriter discussing complex decision making, ambiguity, making judgement calls and person centred care. They then consider how different professions have different conventions in their respective written formats and try their hand at screenwriting.

**Ceramics** The practice of dentistry in common with many other surgical specialities relies on good proprioceptive and haptics skills, trying to make sense of the unseen, learning how much pressure to exert and navigating uncertainty. The students learn the material science of ceramics but this session explores aesthetic ambiguity of ceramics with a practical session at the dental institute led by an expert artist in this craft and myself. The students handle some of the art work before a drawing session and then explore dental enamel tooth loss associated with abrasion, attrition and erosion through working with ceramic clay themselves.

After each session slides are posted onto the student online learning platform just as a reminder of the main focus of the event with some additional references if they wish to follow up any of the points of discussion. Clinical Humanities programmes aim to nurture student professional and personal development through transdisciplinary and cross-faculty informal learning. Through the inherent subjectivity of the Arts and Humanities, we have found that these opportunities afford the time and space within the curriculum for novices to develop new ideas, promote ethical, humane patient care, self-care and improve student higher order, analytical, observational, reflective, and critical thinking skills. Essentially, the students are encouraged to think creatively for themselves, to innovate and to become comfortable with dealing with subjectivity and ambiguity, all rarely addressed in the formal dental curriculum yet fundamental to the complex decision making and leadership that is the reality of clinical practice and to the delivery of holistic patient care. Looking at the students’ written reflections and watching film recordings of the many informal conversations that take place, it is ‘interesting to note how students begin to develop a language to reflect on their professional identity, articulate the skills they were developing and consider what it means to be simultaneously a learner and a practising clinician’ ( Smyth Zahra and Dunton, 2017). This resonates with a previous survey of six professions including dentistry, which identified; observation, reflection, articulation, collaboration and ‘perspective changing’ amongst twelve informal learning processes which the participants felt were important aspects of professional development ( Cheetham and Chivers, 2001). Providing informal learning opportunities in art galleries, cinemas, pottery classes and museums, yet always maintaining clinical relevance, the Clinical Humanities course appears to promote the three characteristics of personal development; ‘pro-activity, critical reflection and creativity’ that make ‘work-based learning more likely to take place’ ( Marsick and Watkins, 1990) thus enhancing student development when they return to the clinic. The relative importance in approaches to informal learning of the ‘individual’s worldview’ ( Swanwick, 2005) and the social dimension, introspection versus conversation has been previously debated by ( Kotzee, 2012). The formal dental curriculum often assumes that students already know how to reflect on their own practice yet conversations with the students themselves reveal that typically dental students entering University with purely science backgrounds are not skilled in this area and feedback to date suggests that encouraging them to reflect on arts and humanities activities where there is no ‘right answer’ and having curriculum time to experiment with ideas which does not impact on patient safety has helped scaffold this fundamental requirement to becoming a capable practitioner. A strong emphasis has been placed on dialogue ( Polyani, 1958) and as has also been suggested, ( Eraut, 1994) engaging in story -telling and narrative through film helps the students articulate their ideas.

The Clinical Humanities course, affording informal learning opportunities away from the usual workplace of the clinic appears to balance the need to develop reflective skills amongst novices who may yet find the process of critical reflection difficult whilst promoting articulation to help make the tacit explicit and aid learning. On entering university often the students, most coming straight from school ‘want trouble-free knowledge’ ( Meyer and Land, 2010), but given that the reality of professional practice is more complex and ambiguous this is not a feasible approach. Providing the students space to explore subjectivity and creativity yet maintain relevance to their clinical practice allows them to ‘learn to talk the talk’ and develop the ‘high self-esteem’ ( Swanwick, 2005) necessary for both their personal and professional development. Making meaning of what it is to be a dentist through informal dialogue based on epistemologies from the humanities helps students construct their own knowledge through problem solving and story-telling and provides the tools for them to cope with ambiguity, understand the importance of the socio-cultural determinants of health, developing not only reflective, advocacy and analytical capabilities, but also vital humanistic skills thus empowering them to not only deal with the complexities that are the reality of everyday clinical practice but also to provide the best person-centred, holistic care to their patients.

## Take Home Messages

• Informal, transformative learning from the Humanities promotes the development of professional autonomy in undergraduate dental students.
• It is fundamental to teach students of the complex realities of real life clinical practice providing them with the necessary skills to develop learning strategies for the entirety of their practising lives.
• Transformative learning models of professional development encourage self-examination and promote construction of new knowledge.
• Humanities embedded in clinical curricula through transdisciplinary, informal learning promote the importance of the subjective, reflexivity, improve advocacy and critical thinking skills and educate for the socio-cultural determinants of health and delivery of person centered care.

## Notes On Contributors

Flora Smyth Zahra is a dentist and Senior Clinical Teacher at King’s College London Dental Institute. She has a degree in English Literature and advocates for more Humanities content in clinical curricula. She is particularly concerned with improving critical analysis skills, educating for ambiguity and complexities in clinical practice and promoting the necessary reflexivity and cutural humility to nurture deep understanding of the socio-cultural determinants of health, inequalities and inequities in access to healthcare and aid the delivery of person-centered care. Humanities epistemologies offer clinical students transformative, informal learning opportunities that support their personal and professional development in these areas. She is a Fellow of the Higher Education Academy, a Council member of the Academy of Medical Educators UK and a project partner of The Collaborating Center for Values-Based practice in Health and Social Care, St Catherine’s College Oxford.
