# Supplementary material for: Clinical Humanities; informal, transformative learning opportunities, where knowledge gained from Humanities epistemologies is translated back into clinical practice, supporting the development of professional autonomy in undergraduate dental students
Source: MedEdPublish (2016). 2018 Nov 14;7:163. Originally published 2018 Aug 7. [Version 2] doi: 10.15694/mep.2018.0000163.2 (PMC10701855; doi:10.15694/mep.2018.0000163.2)
Supplement: Peer review history

# Reviewer report: Reviewer response for version 2

**DOI:** 10.21956/mep.20229.r31341

**Published:** 2019-04-01

**Authors:** Sateesh Babu Arja

**Affiliations:** 1 Avalon University School of Medicine

This review has been migrated. The reviewer awarded 4 stars out of 5 A very well written paper and thoroughly enjoyed reading this manuscript. Humanities is such an important topic in medical education fostering empathy, communication skills, and professional behaviors. The authors have detailed various humanities sessions like Clinical Humanities, History of Medicine and Philosophy, Art, close observation and free writing, Close listening, communication, and performance, Ambiguity in Film, and ceramics. We are also trying to implement humanities in our undergraduate medical program from May 2019. We are including sessions of role-playing, reflective writing, history of medicine, and arts. We are enthusiastic to see the feedback and how students receive the humanities module after implementation. This paper is very useful whoever would like to implement humanities in their educational programs.

Reviewer Expertise:

NA

No decision status is available

# Reviewer report: Reviewer response for version 2

**DOI:** 10.21956/mep.20229.r31340

**Published:** 2018-11-14

**Authors:** P Ravi Shankar

**Affiliations:** 1 American International Medical University

This review has been migrated. The reviewer awarded 5 stars out of 5 I enjoyed reading the first version of the manuscript. As mentioned by the author in the revised second version, a description of the various activities undertaken by the author makes the manuscript more interesting and useful. I would like to congratulate the author on her initiatives. All those interested in the medical/health humanities will find this article to be of interest.

Reviewer Expertise:

NA

No decision status is available

# Reviewer report: Reviewer response for version 2

**DOI:** 10.21956/mep.20229.r31339

**Published:** 2018-11-14

**Authors:** Trevor Gibbs

**Affiliations:** 1 AMEE

This review has been migrated. The reviewer awarded 5 stars out of 5 I thoroughly enjoyed reading this paper first time around and now that the author has been able to add the practical descriptions of what happens to the students, then I enjoyed reading it more and learned a lot. Yes, I do want to sit in on these sessions Flora and maybe some time I will be cheeky enough to!I hope that this piece if research doesn't stop there however- a longitudinal study of these dental students exposed to this Humanities course is very much called for- I would like to see what this early exposure does once they are in the real world and if this new learning leads them to be a "different"- whatever that means-dentist.An essential read for all those involved in developing the Humanities in education.

Reviewer Expertise:

NA

No decision status is available

# Reviewer report: Reviewer response for version 1

**DOI:** 10.21956/mep.19115.r27574

**Published:** 2018-08-07

**Authors:** Trevor Gibbs

**Affiliations:** 1 AMEE

This review has been migrated. The reviewer awarded 3 stars out of 5 An interesting paper, well written and well thought -through in its background element The passion for the Humanities clearly shows in the author's writing and I imagine that this passion is soundly reflected in her teaching- I would like to be a student in this class. In its present form however, I feel that this paper stops at a vital part- that of describing and eventually evaluating the interesting activities that the student participates in - I wanted to read more and although the references guide me to the relevant descriptive paper I feel that these elements could have been added to the paper, if only through describing one or two activities in more detail. Not having these does reduce the value of the paper somewhat.

Reviewer Expertise:

NA

No decision status is available

# Reviewer report: Reviewer response for version 1

**DOI:** 10.21956/mep.19115.r27576

**Published:** 2018-08-07

**Authors:** Hedy S. Wald

**Affiliations:** 1 Warren Alpert Medical School of Brown University

This review has been migrated. The reviewer awarded 3 stars out of 5 Thank you for this interesting and informative article…and for your passion for integrating the humanities within dentistry education. How wonderful. And how valuable for a holistic approach to the transformative process of professional identity formation for providing humanistic patient care. And, as emerging literature is emphasizing, for humanizing and rehumanizing ourselves as caring and compassionate practitioners. The offered perspective is interesting and scholarly.I will echo some of the already posted reviews by offering the perception that this manuscript could be strengthened with inclusion of content that bridges theory to practice. I wanted to hear more about the curriculum innovations, even some brief descriptions within the pilot and would look forward to published outcomes in the future eg. how received? impacting patient care? (or at least perceived as such), impacting wellbeing/engagement? In addition, the manuscript could be strengthened with inclusion of some additional references within the literature review on the topic of professional identity formation (given that this was mentioned in the text). Additional references on past efforts to include the medical humanities in dentistry education would provide helpful background as further context for this author’s initiatives…Some references for consideration: Neidle (1980, J Dental Educ) in an article entitled “Dentistry-Ethics-The Humanities: A Three-Unit Bridge” commented: “Training dentists without providing them with this background is to "run the risk of creating technicians unable to make creative judgments” https://eric.ed.gov/?id=EJ236966...Vergnes et al, 2015 in the J Am Dent Assoc asked “What About Narrative Dentistry?”https://www.ncbi.nlm.nih.gov/pubmed/26025827 and Isaac et al (2015) in the J Dental Educ explored the “Impact of Reflective Writing Assignments on Dental Students’ Views of Cultural Competence and Diversity” http://www.jdentaled.org/content/jde/79/3/312.full.pdfBrett-MacLean et al (2010) in the journal Reflective Practice discussed their approach to using “Film as a means to introduce narrative reflective practice in medicine and dentistry: a beginning story presented in three parts.”

Reviewer Expertise:

NA

No decision status is available

# Reviewer report: Reviewer response for version 1

**DOI:** 10.21956/mep.19115.r27577

**Published:** 2018-08-07

**Authors:** P Ravi Shankar

**Affiliations:** 1 American International Medical University

This review has been migrated. The reviewer awarded 3 stars out of 5 Thank you for the opportunity to review this interesting manuscript. The author has reiterated the various uses of the humanities which have been described in the literature. The competencies at a broader level of different health science students share many similarities. The author could expand on Kennedy’s model of professional development. I would also be interested in the author’s definition of informal learning. The humanities play an important role in professional development as has been well described by the author.I would be interested in the details of the clinical humanities course offered to dental students at the author’s institution. I believe the description has been provided in the article in the British Dental Journal but a brief synopsis will be useful. How was the clinical relevance of various activities during the course maintained? The article will be of particular interest to all educators interested in the humanities in clinical and health education.

Reviewer Expertise:

NA

No decision status is available

# Reviewer report: Reviewer response for version 1

**DOI:** 10.21956/mep.19115.r27575

**Published:** 2018-08-07

**Authors:** David Bruce

**Affiliations:** 1 None

This review has been migrated. The reviewer awarded 2 stars out of 5 the advantage of interactions with peers and teachers and the ability to develop their responses to clinical scenarios. While this is fully explained in the paper, familiarity to the Kennedy model or a at least a read of the Kennedy paper is required to make full sense of this. The paper then moves to the role of clinical humanities and how this might enhance professionalism and personal development of dental students. The case for including medical humanities is well made by review of previous literature on the topic. However, the King’s College pilot project is only partly explained in this paper, and as readers we have to access the authors reference to this work in the British Dental Journal to read more about the pilot and results. I would recommend that readers to do this as the British Dental Journal paper fully explains this work. This paper will be of interest to those involved in clinical humanities or considering introducing humanities to their curricula, but as a non-expert I felt more description of the pilot would have been helpful

Reviewer Expertise:

NA

No decision status is available
